# Lagging Immune Response to *Haemophilus influenzae* Serotype b (Hib) Conjugate Vaccine after the Primary Vaccination with Hib of Infants in The Netherlands

**DOI:** 10.3390/vaccines8030347

**Published:** 2020-06-30

**Authors:** Leo Schouls, Corrie Schot, Richarda M. de Voer, Fiona van der Klis, Mirjam Knol, Irina Tcherniaeva, Guy Berbers

**Affiliations:** 1 Infectious Diseases Research, Diagnostics and laboratory Surveillance (IDS), National Institute for Public Health and the Environment, 3721MA Bilthoven, The Netherlands; Corrie.Schot@rivm.nl; 2 Immunology of Infectious Diseases and Vaccines (IIV), National Institute for Public Health and the Environment, 3721MA Bilthoven, The Netherlands; Richarda.deVoer@radboudumc.nl (R.M.d.V.); Fiona.van.der.Klis@rivm.nl (F.v.d.K.); Irina.Tcherniaeva@rivm.nl (I.T.); Guy.Berbers@rivm.nl (G.B.); 3 Department of Human Genetics, Radboud Institute of Molecular Life Sciences, Radboud University medical center, 6525GA Nijmegen, The Netherlands; 4 Infectious Diseases, Epidemiology and Surveillance (EPI), National Institute for Public Health and the Environment, 3721MA Bilthoven, The Netherlands; Mirjam.Knol@rivm.nl

**Keywords:** Hib, vaccination, reduced immunogenicity, The Netherlands

## Abstract

In 1993, a *Haemophilus influenzae* serotype b (Hib) conjugate vaccine was introduced in the Dutch national immunization program, resulting in a sharp decrease in invasive Hib disease. We used a population-based set of serum samples collected in The Netherlands in 2006–2007 (Pienter-II, 5696 sera) to assess the concentration of antibodies to the capsular polysaccharide of Hib, and compared the results with those obtained from a similar set collected in 1995–1996 (Pienter-I, 7837 sera). Post-primary vaccination serum samples from children aged 6–11 months from the Pienter-II study contained approximately 4-fold lower anti-Hib antibody concentrations than samples from children from the Pienter-I study. No such difference was found in post-booster samples from children older than 11 months of age. In Pienter-II, the proportion of children aged 6–11 months with anti-Hib antibody concentrations below the putative protective concentration of 0.15 µg/mL was 30%, which is significantly higher than in the Pienter-I study (12%). Fewer children in the Pienter-II group developed antibodies able to kill Hib in a serum bactericidal assay compared to the Pienter-I children. The cause of the lagged response in Pienter-II children remain uncertain, but lack of natural boosting, interference by the acellular pertussis vaccine, combining vaccines and acceleration of the schedule may have contributed.

## 1. Introduction

The Netherlands introduced nationwide vaccination against *Haemophilus influenzae* serotype b (Hib) in April 1993 using a 4-dose vaccination schedule at 3, 4, 5 and 11 months of age. Since then the incidence of invasive Hib disease has declined dramatically from 28.7 per 100,000 children aged 0–4 years in 1992 to 0.80 per 100,000 in 2001. However, despite 95% vaccine coverage, a small number of fully vaccinated children in The Netherlands have experienced invasive Hib infection. In 2002, the number of cases of invasive Hib disease in Dutch children aged 0 to 4 years started to increase again, reaching its peak in 2005 with an incidence of 2.57 per 100,000 [1,2,3], which thereafter decreased to 0.97 per 100,000 in 2009. Genotyping of the Hib isolates obtained from the Hib patients did not reveal the emergence of particular genotypes that were capable of explaining the observed increase. No other factors were found that could explain the increase and as a result the cause of the transient resurgence of Hib disease has remained elusive.

The unexpected rise in the incidence of invasive Hib disease emphasizes the importance of post-licensing evaluation of vaccines to assess the efficacy of the vaccination program. Mass vaccination may change the epidemiological dynamics of infectious diseases, e.g., the elimination of circulation of the pathogen may result in reduced natural exposure that may boost immunity. Serological surveillance may be of particular value for diseases like Hib disease with a small number of cases as a result of mass vaccination with high coverage [4]. Furthermore, it offers the opportunity to identify groups within the population in which the vaccination is less effective and may indicate where changes in vaccination strategy are required. In order to estimate the immunity of the Dutch population against pathogens covered by the national immunization program, two large-scale, population-based studies were performed, the first in 1995–1996 and the second in 2006–2007 [5,6]. The serum samples collected during these studies, designated as Pienter-I and Pienter-II, were used to assess the antibody concentrations against virtually all pathogens for which the national immunization program protects, e.g., pneumococci and pertussis.

The aim of this study was to compare the antibody concentrations against Hib polysaccharide and assess the degree of protection against Hib disease in the Dutch population 13 years after the introduction of nationwide Hib vaccination. This revealed a lag in the immune response against the Hib conjugate vaccine after the primary vaccination series in children enrolled in the Pienter-II study.

## 2. Materials and Methods

### 2.1. Study Population

Two independent cross-sectional population-based serosurveillance studies were carried out in The Netherlands. The serum samples for the first study, designated as the Pienter-I study, were collected between October 1995 and December 1996. The samples for the second survey, the Pienter-II study, were collected between February 2006 and June 2007. The study proposal for the Pienter-II survey was approved by the Medical Ethics Testing Committee of the Foundation of Therapeutic Evaluation of Medicines (METC-STEG) in Almere (clinical trial number: ISRCTN 20164309) and all participants provided signed informed consent. Both studies had the same design, which has been described previously [5,6]. The sample set used in the current study is referred to as the national sample (NS) and comprised 7864 serum samples in the Pienter I study and 5702 serum samples in the Pienter II study.

### 2.2. Serology

Hib-specific antibody levels in the samples from the Pienter-I study were reported in 2003 and were assessed by an enzyme-linked immunosorbent assay (ELISA) using purified Hib capsular polysaccharide [3,7,8]. In the current study, we assessed the anti-Hib IgG antibody concentrations in all available serum samples from the Pienter-II survey from children aged 0–23 months (*n* = 399) and from persons aged 2–79 years (*n* = 5299) by a fluorescent-bead-based multiplex immunoassay (MIA) as previously described [9]. In addition, all available samples from the children aged 0–23 months from the Pienter-I study (*n* = 448) and a randomly selected set of samples from the Pienter-I collection from 1404 individuals aged 2–79 years were analyzed by MIA in the current study. Briefly, purified Hib-polysaccharide was coupled to fluorescent beads via a poly-L-lysine linker. In the MIA, a mixture of beads was used to simultaneously detect antibodies against Hib-polysaccharide and meningococcal polysaccharides of serogroups A,C, W-135 and Y. Samples were analyzed using a Bio-Plex system in combination with the Bio-Plex Manager software version 4.1.1 (Bio-Rad Laboratories, Hercules, CA, USA). The median fluorescent intensity was converted to the amount of IgG (µg/mL) by interpolation from a 5-parameter logistic standard curve.

The level of Hib-specific functional antibodies was determined by a serum bactericidal antibody assay (SBA) using baby rabbit complement and the Hib strain Eagan as described before [10]. SBA titers were expressed as the ^2^log of the reciprocal of the final serum dilution yielding 90% killing. If no SBA titer could be measured (no inhibition with serum diluted 1:4, ^2^log < 2) a value of 1 was assigned for statistical purposes. The nature of the bactericidal antibodies, anti-PRP or against other components, was assessed by absorption of the sera samples with purified Hib-polysaccharide (*Haemophilus influenzae* b polysaccharide Polyribosyl ribitol phosphate (PRP) code 02/208, NIBSC, Potters Bar, Hertfordshire, UK). For the SBA, 5 µL serum was mixed with 5 µL of a 1 mg/mL solution of purified Hib-polysaccharide followed by a 1 h incubation at 37 °C for 1 h and overnight incubation at 4 °C.

### 2.3. Statistical Analysis

Data analyses were performed in Prism 8.2.1 (GraphPad, La Jolla, CA, USA). Geometric mean concentrations (GMC) or geometric mean titers (GMT) of Hib-specific IgG and serum bactericidal activity, respectively, were calculated for both surveys in different age-cohorts. The percentage of seroprotection was calculated based on the internationally accepted cut-offs for short term (0.15 µg/mL) and long term seroprotection (1.0 µg/mL [11]. Age-specific differences in Hib IgG levels and SBA titers between the Pienter-I and Pienter-II surveys in each age cohort were determined by a nonparametric two-tailed Mann-Whitney test. Differences were considered statistically significant if *p* < 0.05.

## 3. Results

### 3.1. Hib-Specific IgG Levels in the Pienter-I and Pienter-II Study

**Figure 1:**
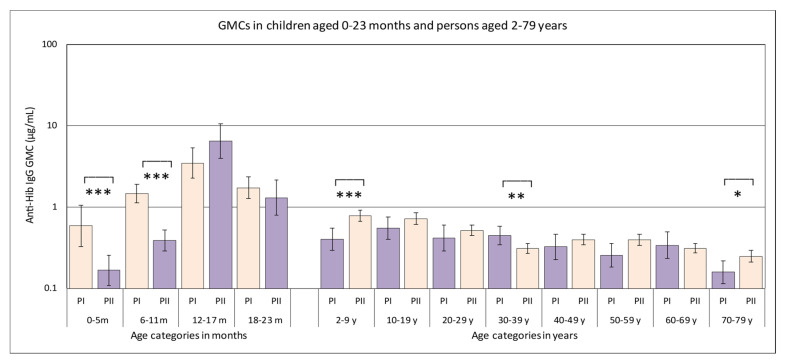
Geometric mean concentrations (GMCs) of antibodies directed against Hib polysaccharide in serum samples from the Pienter-I (salmon colored bars) and Pienter-II (purple bars) studies. Only samples belonging to the national sample were used. The 95% confidence interval of the GMC is displayed as error bars in the figure. The age categories where the GMCs in the Pienter-I and Pienter-II studies are statistically significant different, are marked with asterisks (*** *p*-value < 0.001, ** *p*-value < 0.002, * *p*-value < 0.033.). The *Y*-axis is a logarithmic scale.

Analysis of the serological data of all samples from persons belonging to the national sample, revealed statistically significant differences between the GMCs of sera from the Pienter-II survey compared to those in samples collected during the Pienter-I survey (Figure 1, Table 1). In samples from children younger than 2 years of age, these differences were statistically significant for children aged 0–5 months and those aged 6–11 months, with GMCs of the Pienter-II groups being 3.5-fold and 3.8-fold lower than those of the Pienter-I group, respectively. Stratification of the group aged 0–23 months per month showed that the biggest difference was observed for the samples from the children aged 6 months, where there was a statistically significant 8-fold difference in GMC between the Pienter-I and Pienter-II samples. For the other stratified age groups among the children aged 0–23 months, the numbers were too small and variation in IgG concentrations was too high to obtain statistically significant values. In the serum samples obtained from the 2–79 years group, there were statistically significant differences in the age groups, 2–9 years and 30–39 years. In the 2–9 years age group, the GMC of the samples collected during the Pienter-II survey was 2.0-fold higher than that of the samples from the Pienter-I survey. In the 30–39 years age group, the opposite occurred and the GMC of the Pienter-I samples was 1.4-fold higher than that of the Pienter-II samples.

**Figure 2:**
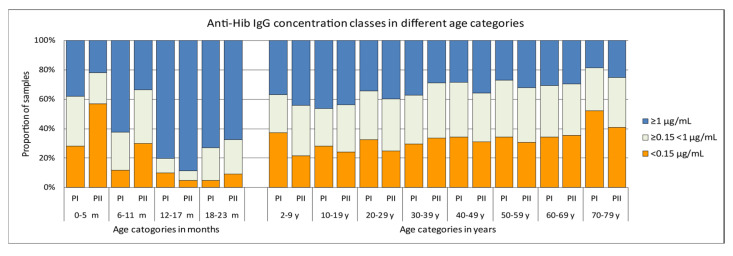
Differences in proportions of antibody concentrations classes in serum samples from the Pienter-I and Pienter-II survey. Only samples belonging to the national sample were used. The figure displays the distribution of samples with antibody concentrations of <0.15 µg/mL, ≥0.15 < 1 µg/mL and ≥1 µg/mL.

**Figure 3:**
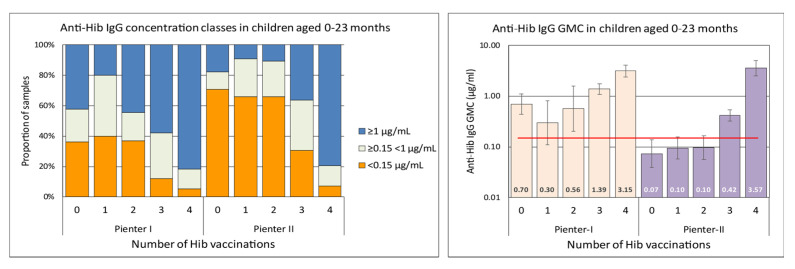
Left panel, differences in proportions of antibody concentrations classes in serum samples from 0–23 month old children from the Pienter-I and Pienter-II survey, stratified by the number of vaccinations they received at time of sampling. Right panel, GMCs of the same serum samples. The GMC values are displayed in the lower part of the bars. The red horizontal line denotes the presumed protective level of 0.15 µg/mL. The *Y*-axis is a logarithmic scale.

In the Pienter-II samples of the 0–5 months age group there was a significantly larger proportion (65/114, 57%) with anti-Hib IgG concentration below the presumed protective level of 0.15 µg/mL than in the Pienter-I group (28%, 14/50) (Figure 2). In the 6–11 months age group there was a similar, although less pronounced difference, with 30% of the Pienter-II and 12% of the Pienter-I samples below the 0.15 µg/mL level. In the 6–11 month age group samples from the Pienter-I survey the proportion of samples with anti-Hib IgG concentrations ≥1 µg/mL was nearly twice as high as in the samples from the Pienter-II survey (62% vs 33%). In the age groups of 2–79 years, no pronounced differences in the proportion between the Pienter-I and Pienter-II samples were seen. Comparing the protective levels in the Pienter-I and Pienter-II cohorts by stratifying the samples based on the number of Hib vaccinations 0–23 month old children received, revealed an even more pronounced difference (Figure 3). In the Pienter-I study, a considerably higher proportion of children already had more protective antibodies before and after the primary vaccinations than their Pienter-II counterparts. Analysis of the GMCs in these groups showed that samples from Pienter-I children who had received one Hib vaccination, had a GMC that was significantly lower than the GMC in children who not yet had received a Hib vaccination. The more vaccinations the Pienter-I children received, the higher the GMCs and the higher the proportion of protected children were. In contrast, GMC in serum samples from the Pienter-II children after one or two Hib vaccinations did not differ from the GMC of the samples of the unvaccinated children. However, sera from Pienter-II children who received three vaccinations had a significantly higher GMC. GMCs of all pre-booster Pienter-I groups were statistically significantly higher than their counterparts from the Pienter-II group. There were no differences in the proportions of protected children and GMCs between both cohorts after the booster vaccination. The Hib vaccine-induced immune response in Pienter-II children appeared to lag, but this delay was nullified by the booster vaccination. 

### 3.2. Hib-Specific Serum Bactericidal Assay

**Figure 4:**
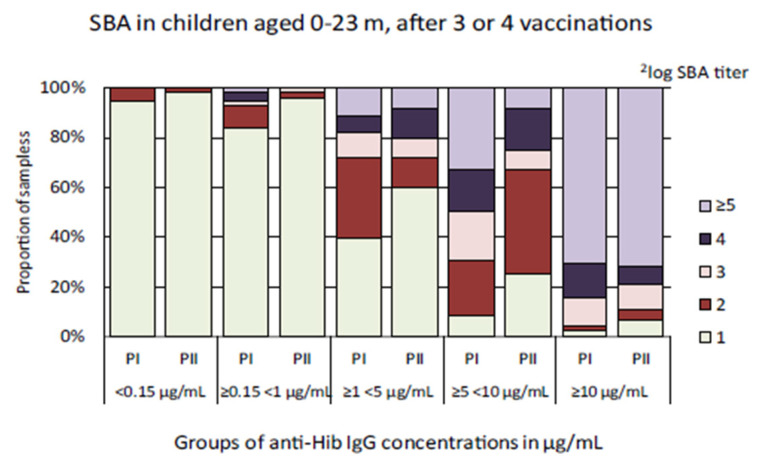
Distribution of ^2^log serum bactericidal antibody (SBA) titers in samples from children aged 0–23 months who received three or four Hib vaccination doses. The serum samples were stratified in groups based on the anti-Hib antibody concentrations found in the multiplex immunoassay (MIA). The light green bar-segment denotes the portion of samples in which no bactericidal activity was detected (^2^log SBA titer = 1).

To assess whether vaccine-induced Hib-specific antibodies in serum samples collected in the Pienter-II study were equally bactericidal as those in samples from the Pienter-I survey, serum samples from children aged 0–23 months who received three or four Hib vaccinations were analyzed in the SBA. To ensure the analyses were not skewed by differences in anti-Hib IgG concentrations, serum samples were divided into groups with similar anti-Hib IgG concentrations for comparison. There was no statistically significant difference between the GMCs of the Pienter-I and Pienter-II groups. The GMTs of the ≥0.15 < 1 µg/mL and ≥5 < 10 µg/mL serum groups were slightly, but statistically significant, higher for the Pienter-I samples than for the Pienter-II samples (Table 2). Inspection of the data at the SBA titer level revealed a considerable difference between the Pienter-I and Pienter-II samples (Figure 4). Of the samples with ≥1 < 5 µg/mL anti-Hib IgG concentrations, 40% of the children enrolled in the Pienter-I did not yield detectable bactericidal activity. In similar samples from children from the Pienter-II study, 60% of the samples did not display bactericidal activity as measured by SBA.

To determine the nature of the bactericidal antibodies, a random selection of samples of the Pienter-I and Pienter-II collection from children 0–23 months of age who had received three or four Hib vaccinations and had ^2^log SBA-titers ≥3 were absorbed with purified Hib-polysaccharide and the decrease in SBA-titer due to the absorption was measured. In the Pienter-I sera 63% (24/38) and in 71% (20/28) of the Pienter-II sera, bactericidal activity could be completely blocked by the absorption Hib polysaccharide. Furthermore, in 13% (5/38) of the Pienter-I sera and in only 4% (1/28) of the Pienter-II sera bactericidal activity could not be blocked by Hib-polysaccharide at all. Remarkably, the latter samples all had ^2^log SBA titers ≥7.

## 4. Discussion

A serosurvey performed on samples collected in 1995–1996 (Pienter-I) showed that vaccination yielded good protection against Hib infection with GMCs ≥ 1 µg/mL IgG against Hib-polysaccharide in children 5–23 months of age [3,12]. The study presented here shows that compared to the previous survey, the immune response to the primary series Hib vaccination is delayed in children aged 0–5 and 6–11 months from the 2006–2007 Pienter-II study, with GMCs nearly 4-fold lower than those found in the Pienter-I study. In the Pienter-I study, 12% of the children did not have antibody levels at or above the putative protective level of 0.15 µg/mL after the primary vaccinations series. In children enrolled in the Pienter-II survey, the fraction of putatively unprotected children was 30%, more than twice as high as observed in the Pienter-I study. Also the proportion of Pienter-II samples from this age group containing antibody concentrations of ≥1 µg/mL was considerably lower (33%) than in the Pienter-I survey (62%). The differences between the proportions of unprotected children and GMCs in both Pienter cohorts correlated with the number of Hib vaccinations the children had received. The GMC in unvaccinated Pienter-I children was much higher than in unvaccinated Pienter-II children, strongly suggesting that Pienter-I children had received maternal anti-Hib antibodies from the mothers who were exposed to naturally circulating Hib in the Pienter-I era. This natural boosting was absent during the Pienter-II collection period as circulation of Hib was virtually non-existent. The presence of maternal antibodies in infants may inhibit the development of the infant’s immune response after vaccination, a phenomenon called blunting. In the Pienter-I group the proportion of children with high antibody concentrations (≥1 µg/mL) and the GMC dropped by a factor of two after the first Hib vaccination. However, during the primary vaccination series, Pienter-I children responded well with gradually increasing GMCs and 90% had protective antibody levels after the third vaccination. In contrast, children in the Pienter-II group were slower to respond and the GMC only increased after the third vaccination with 70% of the children having protective antibody levels. There were no statistically significant differences between GMCs obtained in the two surveys in children that had received a booster vaccination. In individuals aged 2–79 years, the anti-Hib GMCs were low, but above the 0.15 µg/mL seroprotective level. SBA of serum samples from 0–23 months-old children who received three Hib vaccinations revealed a lower SBA-GMT in the samples from the Pienter-II study than from the Pienter-I study. In addition, only 40% of the Pienter-II serum samples with anti-Hib concentrations of ≥1 < 5 µg/mL from children that received three or four vaccinations, were able to kill Hib in the in vitro assay. In contrast, 60% of similar samples from the Pienter-I group killed Hib in the SBA.

Apart from natural boosting, several other reasons may explain the observed lag in the immune response to the Hib vaccination in children from the Pienter-II study. The introduction of an accelerated vaccination schedule may have contributed to the observed slow responses. To reduce the number of cases of pertussis in young children in The Netherlands, the pertussis as well as the 4-dose Hib vaccination schedule of 3, 4, 5 and 11 months of age introduced in 1993, was shifted to start at a younger age in 1999, with doses given at 2, 3, 4 and 11 months. Consequently, this accelerated vaccination schedule was in use when the samples for the Pienter-II study were collected, but not during the collection of samples for the Pienter-I survey. However, several studies have shown that the response against the Hib polysaccharide conjugate vaccine is effective provided that the first dose is not administered before 4–6 weeks of age [13]. In addition, many countries are using the accelerated schedule and have not reported delayed or reduced responses. It therefore seems unlikely that the accelerated schedule has caused the reduced response observed in the Pienter-II study. Alternatively, the introduction of Hib vaccination in a combination vaccine could be the reason for the observed low anti-Hib IgG concentrations after the primary Hib vaccination series. Initially, the Hib vaccine was administered as a separate vaccine in The Netherlands, but since 2003 the Hib vaccine is given combined with diphtheria, tetanus, whole cell pertussis, and inactivated poliovirus (DTwP-IPV-Hib). Combining vaccine components may lead to interference between the components [14,15]. Studies have shown that interactions between concurrently administered polysaccharide conjugate vaccines can have negative as well as positive effects on the antibody responses [14,16,17]. In some studies, vaccination with Neisseria meningitidis serogroup C conjugate vaccine (MenCC) enhanced the response against the Hib polysaccharide [15]. However, during the study period, MenC vaccination in The Netherlands was given at 14 months, and therefore, it probably does not play a role. In 2005, The Netherlands replaced the whole cell pertussis vaccine with an acellular pertussis vaccine administered to infants in a combination vaccine together with Hib and the other components (DTaP-IPV-Hib). In 2006, The Netherlands changed the vaccine composition again, replacing the 3-component (Infanrix, GSK, Rixensart, Belgium) acellular pertussis vaccine by a 5-component vaccine (Pediacel, Sanofi Pasteur MSD, Maidenhead, UK). All of the 0–23 month old individuals in the Pienter-II study received DTaP-IPV-Hib vaccine for the primary series, and 85% of the children eligible for a booster vaccination also received DTaP-IPV-Hib as the booster. In contrast, the Hib vaccination was given as a separate vaccine during the collection of samples for the Pienter-I survey. The concurrent use of the acellular pertussis vaccine has been associated with reduced Hib immunogenicity [18,19]. Researchers in the United Kingdom deduced that interference by the acellular pertussis vaccine was responsible for a considerable increase in the number of Hib vaccine failures in 2002, which occurred mainly in children 1–4 years of age [20]. This increase in vaccine failures led to a catch-up campaign in 2003 and the introduction of a Hib booster vaccination at the age of 12 months in 2006. Southern et al. [21] showed that children who received a combination vaccine containing acellular pertussis vaccine developed significantly lower anti-PRP antibody titers compared to children who received a combination vaccine containing whole cell pertussis vaccine. However, after receiving a booster dose, there was no longer a statistical difference between the antibody concentrations induced by both combination vaccines. The resemblance between these observations and the results that we obtained is striking. In The Netherlands the Hib vaccination schedule has always included a booster vaccination at 11 months of age and this would explain why The Netherlands did not experience an increase in the number of Hib vaccine failures like the United Kingdom did. A recent study by Collins et al. on Hib seroprevalence in England and Wales in 2013–2014, showed that the median anti-Hib antibody concentration in 6–11 month old children was just above 1 µg/mL and 88% of the children had anti-Hib antibody concentrations above 0.15 µg/mL [22]. In this pre-booster age group, 50% of the children had anti-Hib antibody concentrations ≥1 µg/mL. These results closely resemble those obtained in our study for the Pienter-II cohort. During our large RCT on pneumococcal vaccination in 2013, we also tested anti-Hib responses pre- and post-booster [22]. This study corroborated the observed low pre-booster anti-Hib antibody concentrations found in the Pienter-II cohort and the sharp increase after the booster took place.

For the 2–9 years and 10–19 years age groups, the GMCs of the anti-Hib IgG antibodies in the Pienter-II samples were higher than those for the samples from the Pienter-I survey. The most likely explanation for this difference is the fact that 95% of the children aged 2–9 years had received a full 4-dose Hib vaccination, whereas this was the case for only 15% of the 2–9 year-olds in the Pienter-I group. In the 30–39 years age group, samples collected during the Pienter-I survey had a slight, but statistically significant, higher GMC than those collected for the Pienter-II survey. The reason for this difference remains unexplained, but may be the result of silent boosting. 

Analyses by SBA showed that, despite similar anti-Hib IgG concentrations, a smaller fraction of the Pienter-II serum samples from 0–23 months-old children who received three or four vaccinations were able to kill Hib in an in vitro assay compared to the Pienter-I samples. In a number of serum samples, the bactericidal antibodies could only be blocked partially or not at all using adsorption with purified PRP in the SBA. This may indicate that not all anti-Hib bactericidal antibodies are directed against the polysaccharide, but against other components of Hib. Remarkably, this phenomenon was predominantly seen in serum samples from the Pienter-I study. This suggests that exposure to components other than the capsular polysaccharide of circulating Hib may have induced the latter antibodies. 

Despite the lag in the immune response induced by the Hib vaccine it is clear that the number of cases of Hib disease in The Netherlands is still very low. Near elimination of Hib circulation due to herd immunity is the most likely reason for the continued low incidence of invasive Hib disease. Prior to the introduction of Hib vaccination, the highest incidence of Hib disease was in 6–11 months-old children and in this study, this was the age group that may be less well protected due to a suboptimal response to Hib vaccination. This emphasizes the need to remain vigilant and maintain the surveillance for Hib disease.

## 5. Conclusions

In conclusion, this population based study shows that the response to Hib primary vaccinations in a time period nearly 15 years after the introduction of nationwide Hib vaccination is slow. Compared to a samples collected in a previous serosurveillancestudy two years after introduction of Hib vaccination, responses were lagging, with approximately 4-fold lower anti-Hib antibody concentrations after the primary vaccination series. As a result, the proportion of children of the current study with anti-Hib antibody concentrations below the putative protective concentration of 0.15 µg/mL after the primary vaccination series was three times as high as those in children enrolled in the previous study. In addition, antibodies in children in the current study seemed to be less potent to kill Hib in a serum bactericidal assay. The reason for the lagged response to Hib vaccination remains uncertain, but the lack of natural boosting by circulating Hib may be the most likely explanation. However, interference by the acellular pertussis vaccine component in the combination vaccines, combining vaccines and acceleration of the schedule may have also contributed to the lagging response.

## Figures and Tables

**Table 1:** GMCs in µg IgG/mL of samples from the Pienter-I and Pienter-II surveys stratified by age. The asterisks (*) denote categories where the GMCs of the Pienter-I and Pieter II samples were statistically different.

Age Category	Pienter I			Pienter II		
	*n*	GMC	95% CI	*n*	GMC	95% CI
0–5 month ***	50	0.59	0.33–1.05	114	0.17	0.11–0.25
6–11 month ***	201	1.48	1.14–1.92	181	0.39	0.29–0.52
12–17 month	112	3.48	2.26–5.36	61	6.51	4.01–10.56
18–23 month	85	1.73	1.28–2.34	43	1.31	0.80–2.14
2–9 year ***	212	0.40	0.30–0.55	680	0.78	0.67–0.92
10–19 year	225	0.55	0.40–0.75	694	0.72	0.61–0.85
20–29 year	129	0.42	0.29–0.60	693	0.52	0.45–0.60
30–39 year **	224	0.44	0.34–0.58	705	0.31	0.27–0.36
40–49 year	168	0.32	0.23–0.46	630	0.40	0.34–0.46
50–59 year	171	0.26	0.18–0.36	628	0.39	0.34–0.46
60–69 year	131	0.34	0.23–0.49	721	0.31	0.27–0.36
70–79 year *	144	0.16	0.11–0.22	548	0.25	0.21–0.29

*** *p*-value < 0.001, ** *p*-value < 0.002, * *p*-value < 0.033.

**Table 2:** GMCs (µg IgG/mL) and geometric mean titers (GMT) in (^2^log SBA titer) of samples from the Pienter-I and Pienter-II surveys from children 0–23 months who received three or four Hib vaccination doses.

Anti-Hib IgG Concentration Range	Study	*n*	GMC	95% CI	GMT	95% CI
<0.15 µg/mL	Pienter-I	18	0.05	0.04–0.08	1.04	0.96–1.13
	Pienter-II	48	0.04	0.03–0.06	1.02	0.99–1.04
≥0.15 < 1 µg/mL *	Pienter-I	56	0.38	0.33–0.44	1.17	1.06–1.30
	Pienter-II	65	0.39	0.34–0.45	1.04	0.99–1.09
≥1 < 5 µg/mL	Pienter-I	78	2.28	2.08–2.51	1.87	1.63–2.14
	Pienter-II	50	2.19	1.90–2.51	1.63	1.35–1.97
≥5 <10 µg/mL *	Pienter-I	36	7.08	6.64–7.54	3.25	2.70–3.92
	Pienter-II	12	6.83	5.90–7.93	2.19	1.47–3.27
≥10 µg/mL	Pienter-I	44	18.81	15.65–22.61	5.21	4.56–5.96
	Pienter-II	28	23.34	18.58–29.32	4.66	3.78–5.75

* Statistically significant difference between GMTs (Mann Whitney, *p* < 0.05).
